# Internet gaming disorder and depression mediated by impaired resilience and sleep distress: a three-wave longitudinal study among Chinese adolescents

**DOI:** 10.1017/S2045796025000046

**Published:** 2025-02-19

**Authors:** P. Peng, Z. M. Chen, S. L. Ren, Y. He, J. G. Li, A. J. Liao, L. L. Zhao, X. Shao, S. S. Chen, R. N. He, Y. D. Liang, Y. G. Tan, X. G. Chen, Y. H. Liao, J. S. Tang

**Affiliations:** 1 Department of Psychiatry, Sir Run Run Shaw Hospital, Zhejiang University School of Medicine, Hangzhou, ZJ, China; 2 Department of Psychiatry, Zigong Mental Health Center, Zigong, SC, China; 3 Department of Nursing, Sichuan Vocational College of Health and Rehabilitation, Zigong, SC, China; 4 Department of Psychiatry, National Clinical Research Center for Mental Disorders, and National Center for Mental Disorders, The Second Xiangya Hospital of Central South University, Changsha, HN, China

**Keywords:** adolescents, depression, internet gaming disorder, resilience, sleep distress

## Abstract

**Aims:**

While the cross-sectional relationship between internet gaming disorder (IGD) and depression is well-established, whether IGD predicts future depression remains debated, and the underlying mechanisms are not fully understood. This large-scale, three-wave longitudinal study aimed to clarify the predictive role of IGD in depression and explore the mediating effects of resilience and sleep distress.

**Methods:**

A cohort of 41,215 middle school students from Zigong City was assessed at three time points: November 2021 (T1), November 2022 (T2) and November 2023 (T3). IGD, depression, sleep distress and resilience were measured using standardized questionnaires. Multiple logistic regression was used to examine the associations between baseline IGD and both concurrent and subsequent depression. Mediation analyses were conducted with T1 IGD as the predictor, T2 sleep distress and resilience as serial mediators and T3 depression as the outcome. To test the robustness of the findings, a series of sensitivity analyses were performed. Additionally, sex differences in the mediation pathways were explored.

**Results:**

(1) IGD was independently associated with depression at baseline (T1: adjusted odds ratio [AOR] = 4.76, 95% confidence interval [CI]: 3.79–5.98, *p* < 0.001), 1 year later (T2: AOR = 1.42, 95% CI: 1.16–1.74, *p* < 0.001) and 2 years later (T3: AOR = 1.24, 95% CI: 1.01–1.53, *p* = 0.042); (2) A serial multiple mediation effect of sleep distress and resilience was identified in the relationship between IGD and depression. The mediation ratio was 60.7% in the unadjusted model and 33.3% in the fully adjusted model, accounting for baseline depression, sleep distress, resilience and other covariates. The robustness of our findings was supported by various sensitivity analyses; and (3) Sex differences were observed in the mediating roles of sleep distress and resilience, with the mediation ratio being higher in boys compared to girls.

**Conclusions:**

IGD is a significant predictor of depression in adolescents, with resilience and sleep distress serving as key mediators. Early identification and targeted interventions for IGD may help prevent depression. Intervention strategies should prioritize enhancing resilience and improving sleep quality, particularly among boys at risk.

## Introduction

Depression is among the most prevalent and challenging mental health issues during adolescence. Globally, approximately 34% of adolescents experience depressive symptoms, and 8% are diagnosed with major depressive disorder (Shorey *et al.*, 2022). Depression is the leading cause of illness and disability in this age group, and its prevalence has alarmingly increased over the past two decades – from 24% between 2001 and 2010 to 37% between 2011 and 2020 (Shorey *et al.*, 2022). This trend underscores the urgent need to understand the factors contributing to adolescent depression, which is essential for developing effective prevention and intervention strategies.

### Internet gaming disorder as a potential risk factor for depressive symptoms

Digital addictions, including internet gaming disorder (IGD), smartphone addiction, internet addiction and social media addiction, are regarded as emerging risk factors for adolescent depression (Chen *et al.*, 2020a; Düll *et al.*, 2024; Karakose *et al.*, 2023). Among these, IGD – characterized by excessive and problematic gaming that causes distress and functional impairment – has been one of the most extensively studied (Düll *et al.*, 2024). Numerous cross-sectional studies have consistently shown a positive association between IGD and depression, with a moderate effect size of 0.33 (Alimoradi *et al.*, 2024; Richard *et al.*, 2020; Yoon *et al.*, 2021). Meta-analyses have also revealed a high comorbidity rate of depressive disorder among individuals with IGD, approximately 34% (Ostinelli *et al.*, 2021).

While cross-sectional studies have established a strong link between IGD and depression, the question of whether IGD predicts future depressive symptoms remains debated. A recent systematic review of cohort studies revealed mixed results: six studies supported IGD as a predictor of depressive symptoms, while seven found no such relationship (Düll *et al.*, 2024). Longitudinal studies in Chinese adolescents have shown similar inconsistencies: several studies during the COVID-19 pandemic found that IGD was associated with worsening depressive symptoms (Chen *et al.*, 2022a, 2022b; Chen *et al.*, 2021), while other studies suggested that IGD may be an outcome, rather than a predictor, of depression among adolescents (Chang *et al.*, 2022a; Teng *et al.*, 2021; Yin *et al.*, 2023). Moreover, few studies have explored the mechanisms through which IGD may lead to depression, highlighting the need for large-scale longitudinal research to clarify this association and identify key mediating factors.

### Impaired resilience and sleep distress as potential mediators between IGD and depressive symptoms

Resilience, a well-known protective factor against mental distress (Watters *et al.*, 2023), may serve as a crucial mediator between IGD and depression. Although longitudinal evidence on the relationship between resilience and IGD is limited, cross-sectional studies consistently observed impaired resilience in individuals with IGD (Canale *et al.*, 2019; Liu *et al.*, 2024; Ma *et al.*, 2024). Additionally, longitudinal research on other behavioural addictions, such as mobile phone and internet addiction, has shown that these addictions can weaken resilience (Li *et al.*, 2022; Shang *et al.*, 2024; Yao *et al.*, 2024). These studies also confirm that reduced resilience mediates the relationship between these addictions and mental health issues, including depression and eating disorders (Li *et al.*, 2022; Shang *et al.*, 2024). Given this evidence, it is plausible that IGD similarly diminishes resilience, making adolescents more vulnerable to depression by weakening their ability to cope with the emotional and psychological challenges. However, no prior studies have specifically tested the mediating role of resilience in the relationship between IGD and depression.

Sleep distress is another potential mediator linking IGD to depression. Longitudinal studies have consistently reported the predictive role of digital addictions on subsequent sleep distress (Byeon *et al.*, 2022; Chang *et al.*, 2022b; Saffari *et al.*, 2023). In addition, recent research suggests that sleep distress may be a common pathway linking those addictions to mental health problems. For example, Li *et al.* found that insomnia mediated 60.6% of the relationship between internet addiction and depression and 44.8% of the relationship between social media addiction and depression among adolescents (Li *et al.*, 2017). Similarly, sleep distress has also been shown to mediate the relationship between smartphone addiction and depression in college students (Liu and Lu, 2022; Zou *et al.*, 2019). In the context of IGD, studies have consistently reported the mediating role of sleep problems between IGD and various negative outcomes, such as psychotic experiences, violence and suicidality (Bersani *et al.*, 2022; Fekih-Romdhane *et al.*, 2023; Garg *et al.*, 2023; Liao *et al.*, 2024). However, only two studies have investigated the relationship between IGD, depression and sleep distress, and both were cross-sectional (Alshammari *et al.*, 2022; Li *et al.*, 2019). More longitudinal research is needed to confirm this mediation effect.

### The potential chain mediating role of sleep distress and resilience

Previous longitudinal studies have demonstrated a link between sleep distress and resilience, indicating that sleep problems may lead to impaired resilience over time (Li *et al.*, 2024; Yao *et al.*, 2024). Consequently, it is plausible that the relationship between IGD and depression could be sequentially mediated, first through sleep distress and subsequently through resilience. In addition to the single mediating roles of each factor, this potential chain mediation may also impact the IGD-depression association.

### The current study

To address these gaps, the present study was conducted to test the following hypotheses: (1) IGD predicts future depression in adolescents, and (2) there is a serial multiple mediation effect of sleep distress and resilience on the association between IGD and depression. Given the known sex differences in both IGD and depression (Marraudino *et al.*, 2022), we also conducted exploratory subgroup analyses based on gender.

## Methods

### Study procedure and participants

This three-wave, school-based longitudinal study was conducted in Zigong, a city in southwest Sichuan, China. The baseline assessment took place in November 2021, involving 44,270 students from grades 7 and 10 across 166 middle schools, recruited using a cluster sampling method. After excluding invalid responses due to excessively short response times, logical inconsistencies (e.g., abnormal age) and incorrect identification information (e.g., erroneous student IDs), the final baseline sample included 41,215 students, reflecting a response rate of 93.1%. Participants were re-assessed in November 2022 (T2) and November 2023 (T3).

At each survey, participants completed the electronic survey during class hours in the computer centre of their respective schools. Before the study commenced, head teachers and psychological teachers received training on the study procedures and measurement tools. They introduced the study’s purpose, supervised the survey administration and answered any questions from the participants.

Participants were informed that their involvement in the study was entirely voluntary, with the option to withdraw at any point. Written informed consent was obtained from all participants, and for those under 18, consent was also secured from their parents. The study protocol was reviewed and approved by the Ethics Committee of the Zigong Mental Health Center (Approval No. 2021003).

### Measurements

#### Internet gaming disorder

IGD was assessed in T1. Participants were first asked if they had engaged in gaming within the past 12 months. Those who answered ‘yes’ were assessed using the Internet Gaming Disorder Scale-Short Form (IGDS9-SF) (Pontes and Griffiths, 2015), which includes nine items based on the DSM-5 criteria for IGD. The IGDS9-SF scores range from 9 to 45, with 32 or above indicating IGD (Qin *et al.*, 2020). Participants who did not engage in gaming were assigned a minimum score of 9 for subsequent analyses. The Chinese version of IGDS9-SF has demonstrated nice psychometric properties (Chen *et al.*, 2020b). The Cronbach’s alpha of IGDS9-SF was 0.913.

#### Depression

Depression was assessed at T1, T2 and T3 using the 9-item Patient Health Questionnaire (PHQ-9), which measures depressive symptoms over the preceding 2 weeks (Costantini *et al.*, 2021). Each item is scored on a 4-point scale from 0 (Not at all) to 3 (Nearly every day), with total scores ranging from 0 to 27. A score of 10 or higher was considered indicative of moderate to severe depression (Levis *et al.*, 2019). The Chinese version of PHQ-9 has demonstrated excellent validity and reliability (Zhang *et al.*, 2013). In the present study, the Cronbach’s alpha of PHQ-9 was 0.898 at T1, 0.907 at T2 and 0.905 at T3.

#### Resilience

We measured resilience at T1 and T2 using the Chinese version of the 10-item Connor–Davidson Resilience Scale (CD-RISC-10), which assesses an individual’s capacity to cope with adversity (Campbell-Sills and Stein, 2007; Ye *et al.*, 2017). The total scores range from 0 to 40. Participants scoring below 26 were categorized into the low resilience group (Ye *et al.*, 2017). The Cronbach’s alpha of CD-RISC 10 was 0.947 at T1 and 0.955 at T2.

#### Sleep distress

The Chinese version of the Pittsburgh Sleep Quality Index (PSQI) was used to assess sleep distress at T1 and T2 (Buysse *et al.*, 1989; Tsai *et al.*, 2005). It consists of 19 items across 7 components, yielding a global score between 0 and 21. A global score above 5 indicated significant sleep distress (Peng *et al.*, 2023). The Cronbach’s alpha of PSQI was 0.871 at T1 and 0.890 at T2 for its seven components.

#### Covariates

We collected demographic and lifestyle data at baseline, including sex, age, school type, residence, alcohol and smoking usage, family structure, left-behind status, only-child status and parental education levels. Anxiety was measured using the 2-item Generalized Anxiety Disorder questionnaire (GAD-2) (Plummer *et al.*, 2016). GAD-2 scores range from 0 to 6, with a score of 3 or above indicating the presence of anxiety (Plummer *et al.*, 2016).

### Statistical analysis

Continuous variables were summarized using means and standard deviations, while categorical variables were presented as frequencies and percentages. Baseline characteristics between adolescents with and without depression were compared using Chi-square tests and Student’s *t*-tests. To evaluate the association between baseline IGD and depression at both baseline and follow-up, univariate and multivariate logistic regression analyses were conducted.

Mediation analysis used T1 IGDS9-SF scores as predictors, T2 PSQI and CD-RISC-10 scores as serial mediators and T3 PHQ-9 scores as outcomes. Missing data were handled using full information maximum likelihood methods. This approach utilizes all available information in the dataset, even when some variables are missing for certain cases, and is known to produce less biased and more efficient estimates compared to listwise deletion or simpler imputation methods (Niu, 2024). However, this method does not include cases where participants did not complete any follow-up assessments. Baseline demographics, anxiety, sleep distress, depression and resilience were controlled as covariates. Bootstrapping (5000 samples) was used to estimate 95% confidence intervals (CIs) for the path coefficients.

We conducted a series of sensitivity analyses to assess the robustness of our findings: (1) re-analysing the data by including only participants with complete data across all three waves (*n* = 28,916); (2) re-analysing the data by including only baseline gamers (*n* = 28,558); (3) re-analysing the model by treating IGD, sleep problems, resilience and depression as a binary variable and (4) removing the sleep-related item from the PHQ-9 to minimize shared variance between the PHQ-9 and PSQI.

Given the well-established sex difference in IGD and depression (Marraudino *et al.*, 2022), we also performed subgroup analysis using multiple-group analysis. Chi-square difference tests were performed to compare an unconstrained model with models where the main paths (IGD → Resilience, IGD → Sleep Distress, Resilience → Depression, Sleep Distress → Depression, Sleep Distress → Resilience and IGD → Depression) were constrained to be equal across sex. Baseline covariates were controlled in both models.

Descriptive analysis, group comparisons and logistic regression were performed using R (version 4.20). Mediation analysis was conducted based on path analysis using Mplus (version 8.1). All mediation models in our study (both unadjusted and adjusted) were saturated. Consequently, model fit indices were not reported. Statistical significance was set at *p* < 0.05 for all two-tailed tests.

## Results

### Sample characteristics

Of the 41,215 students assessed at baseline (T1), 33,479 (81.2%) were reassessed at T2, and 31,523 (76.5%) completed the survey at T3. Overall, 89% of participants completed at least one follow-up. The mean age at baseline was 13.51 years (SD = 1.55), with 49.4% of the participants being boys. Detailed characteristics at T1, T2 and T3 are presented in Table 1. Participants who were boys, in junior middle school, left-behind children, smokers, those from single-parent or remarried families, those with lower parental education or those with impaired resilience, sleep distress or depression at baseline were slightly more likely to drop out (all *p* < 0.05).

**Table 1.** Baseline characteristics of participants of T1, T2 and T3

Variables	T1 (*n* = 41,215)	T2 (*n* = 33,479)	T3 (*n* = 31,523)	Statistic	*P*
**Age, mean ± SD**	13.51 ± 1.55	13.55 ± 1.53	13.44 ± 1.54	*F* = 44.82	<0.001
**School type, *n* (%)**				*χ*^2^ = 72.86	<0.001
Junior middle school	25,341 (61.48)	19,828 (59.23)	18,739 (59.45)		
Senior high school	10,594 (25.70)	8931 (26.68)	8179 (25.95)		
Vocational school	5280 (12.81)	4720 (14.10)	4605 (14.61)		
**Sex, *n* (%)**				*χ*^2^ = 9.14	0.01
Boys	20,372 (49.43)	16,176 (48.32)	15,420 (48.92)		
Girls	20,843 (50.57)	17,303 (51.68)	16,103 (51.08)		
**Only child, *n* (%)**				*χ*^2^ = 0.57	0.751
No	31,034 (75.30)	25,143 (75.10)	23,748 (75.34)		
Yes	10,181 (24.70)	8336 (24.90)	7775 (24.66)		
**Left behind child, *n* (%)**				*χ*^2^ = 6.51	0.039
No	29,059 (70.51)	23,890 (71.36)	22,338 (70.86)		
Yes	12,156 (29.49)	9589 (28.64)	9185 (29.14)		
**Residence, *n* (%)**				*χ*^2^ = 53.21	<0.001
Country	26,376 (64.00)	20,558 (61.41)	19,768 (62.71)		
City	14,839 (36.00)	12921 (38.59)	11,755 (37.29)		
**Drinking, *n* (%)**				*χ*^2^ = 5.55	0.063
No	32,617 (79.14)	26,632 (79.55)	25,168 (79.84)		
Yes	8598 (20.86)	6847 (20.45)	6355 (20.16)		
**Smoking, *n* (%)**				*χ*^2^ = 53.39	<0.001
No	38,046 (92.31)	31,188 (93.16)	29,530 (93.68)		
Yes	3169 (7.69)	2291 (6.84)	1993 (6.32)		
**Family structure, *n* (%)**				*χ*^2^ = 6.58	0.037
Nuclear	32,194 (78.11)	26,392 (78.83)	24,808 (78.70)		
Single-parent or remarried	9021 (21.89)	7087 (21.17)	6715 (21.30)		
**Father’s education level, *n* (%)**				*χ*^2^ = 7.77	0.021
Below high school	31,294 (75.93)	25,125 (75.05)	23,814 (75.54)		
High school or above	9921 (24.07)	8354 (24.95)	7709 (24.46)		
**Mother’s education level, *n* (%)**				*χ*^2^ = 5.39	0.068
Below high school	32,277 (78.31)	25,983 (77.61)	24,607 (78.06)		
High school or above	8938 (21.69)	7496 (22.39)	6916 (21.94)		
**Impaired resilience, *n* (%)**				*χ*^2^ = 17.11	<0.001
No	19,332 (46.91)	15,942 (47.62)	15,273 (48.45)		
Yes	21,883 (53.09)	17,537 (52.38)	16,250 (51.55)		
**Sleep distress, *n* (%)**				*χ*^2^ = 20.49	<0.001
No	29,485 (71.54)	24,008 (71.71)	23,003 (72.97)		
Yes	11,730 (28.46)	9471 (28.29)	8520 (27.03)		
**Depression, *n* (%)**				*χ*^2^ = 18.62	<0.001
No	34,502 (83.71)	28,214 (84.27)	26,759 (84.89)		
Yes	6713 (16.29)	5265 (15.73)	4764 (15.11)		
**Anxiety, *n* (%)**				*χ*^2^ = 11.23	0.004
No	36,641 (88.90)	29,859 (89.19)	28,269 (89.68)		
Yes	4574 (11.10)	3620 (10.81)	3254 (10.32)		
**IGD, *n* (%)**				*χ*^2^ = 0.66	0.719
No	40,535 (98.35)	32,902 (98.28)	30,986 (98.30)		
Yes	680 (1.65)	577 (1.72)	537 (1.70)		

### Prevalence and association of depression with IGD, low resilience and sleep distress

**Figure 1.**
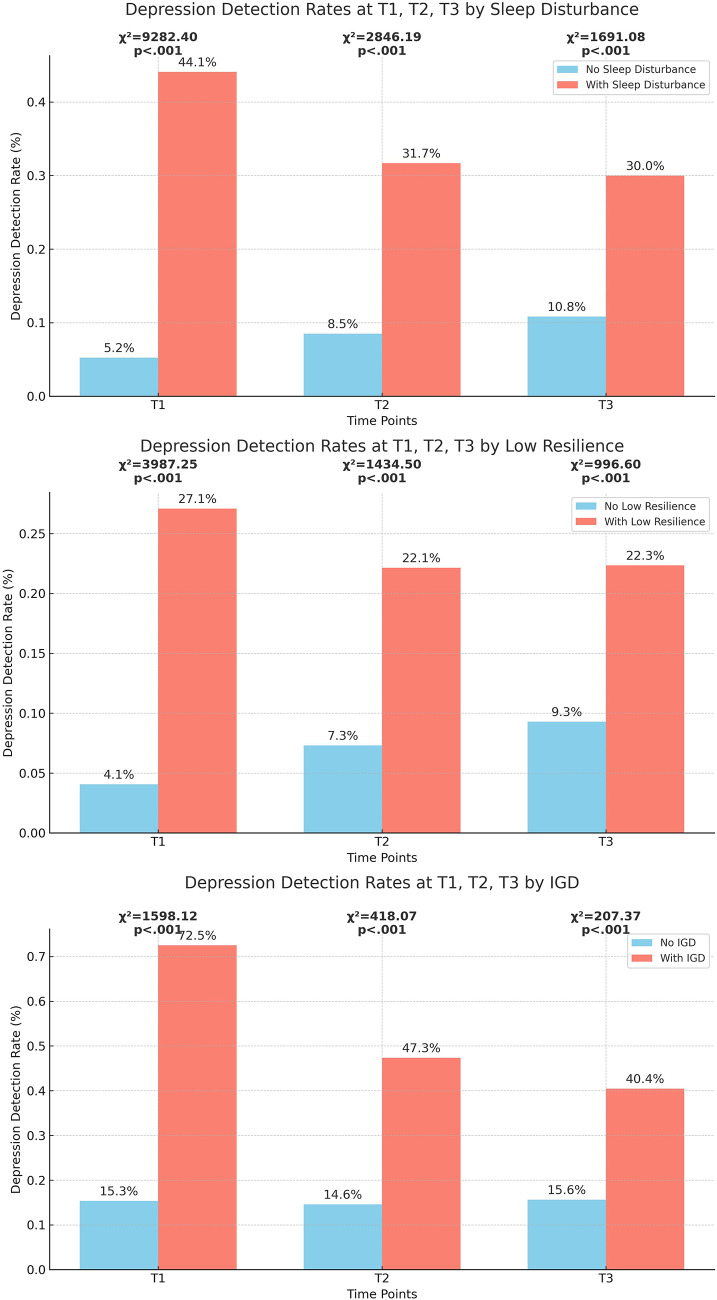
Association of IGD, resilience, sleep distress at T1 with depression at T1, T2, T3.

The prevalence of depression was 16.3% at T1, 15.1% at T2 and 16.0% at T3. As shown in Fig. 1, adolescents with sleep distress, low resilience and IGD consistently exhibited higher rates of depression across T1, T2 and T3 compared to their peers (all *p* < 0.001). For instance, at T1, the depression rate was 72.5% among those with IGD, compared to 15.3% among those without IGD. This pattern persisted, with depression rates at T2 being 47.3% for those with IGD versus 14.6% for those without IGD, and at T3, 40.4% versus 15.6%.

Logistic regression analyses confirmed that low resilience, sleep distress and IGD at T1 were significantly associated with higher odds of depression at T1, T2 and T3 (Table 2). In the unadjusted models, IGD was strongly associated with depression, with odds ratios (ORs) of 14.54 (95% CI: 12.26–17.25) at T1, 5.27 (95% CI: 4.42–6.29) at T2 and 3.66 (95% CI: 3.03–4.41) at T3 (all *p* < .001). After adjusting for baseline characteristics, the association between IGD and depression remained significant but attenuated, with ORs of 4.76 (95% CI: 3.79–5.98, *p* < 0.001) at T1, 1.42 (95% CI: 1.16–1.74, *p* < 0.001) at T2 and 1.24 (95% CI: 1.01–1.53, *p* = 0.042) at T3. Similar trends were observed for sleep distress and low resilience, which also remained significant predictors of depression across all time points.

**Table 2.** Depression at T1, T2 and T3 in relationship to T1 resilience, sleep distress and IGD

	Depression at T1		Depression at T2		Depression at T3	
Characteristics	Unadjusted model	Adjusted model^a^	Unadjusted model	Adjusted model^b^	Unadjusted model	Adjusted model^b^
**Low resilience**	8.76 (8.11–9.47)***	3.95 (3.61–4.33)***	3.61 (3.37–3.87)***	1.76 (1.62–1.90)***	2.81 (2.63–3.00)***	1.66 (1.54–1.79)***
**Sleep distress**	14.27 (13.40–15.20)***	6.17 (5.73–6.64)***	4.98 (4.68–5.30)***	1.94 (1.79–2.09)***	3.52 (3.31–3.75)***	1.67 (1.55–1.80)***
**IGD**	14.54 (12.26–17.25)***	4.76 (3.79–5.98)***	5.27 (4.42–6.29)***	1.42 (1.16–1.74)***	3.66 (3.03–4.41)***	1.24 (1.01–1.53)*

a Adjusted for baseline demographics (age, sex, family type, residence, family structure, parental education level, left-behind status, only-child status, drinking and smoking) and anxiety symptoms.

b Adjusted for baseline demographics (age, sex, family type, residence, family structure, parental education level, left-behind status, only-child status, drinking and smoking), depression and anxiety symptoms.

*** *p* < 0.001, *p < 0.5.

### Mediation analysis

**Figure 2.**
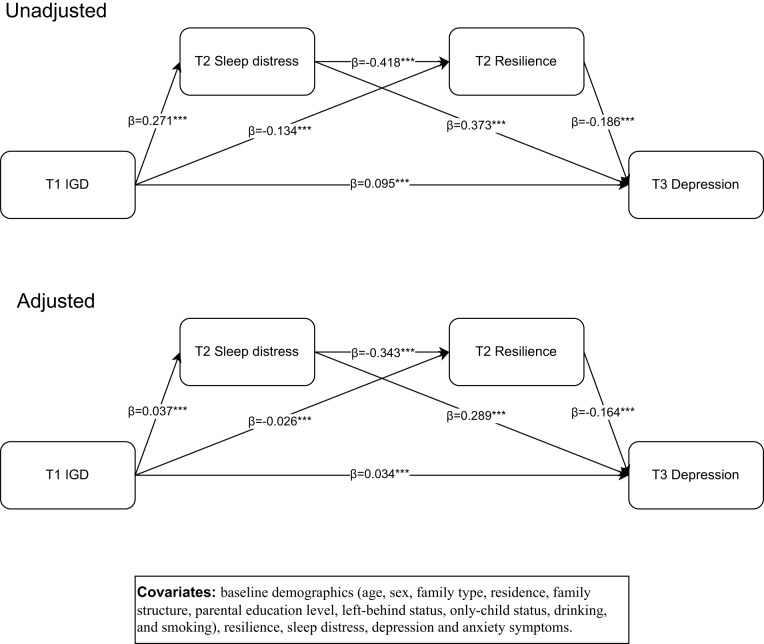
The unadjusted and adjusted mediation model.

Table 3 and Fig. 2 present the results of the unadjusted and adjusted mediation analyses. In the unadjusted model, baseline IGD was a significant predictor of both resilience (*β* = −0.134, 95% CI = −0.144 to −0.124) and sleep distress at T2 (*β* = 0.271, 95% CI = 0.259–0.282), both of which were significant predictors of depression at T3. The path linking sleep distress to impaired resilience was also significant (*β* = −0.418, 95% CI = −0.427 to −0.408). Additionally, IGD at T1 exhibited a direct effect on depression at T3 (*β* = 0.095, 95% CI = 0.082–0.107).

**Table 3.** The mediation effect of sleep distress and resilience between IGD and depression

	Unadjusted		Adjusted	
	*β* (95% CI)	Mediation ratio	*β* (95% CI)	Mediation ratio
**Direct effect (IGD → Depression)**	0.095 (0.082–0.107)	-	0.034 (0.021–0.048)	-
**Indirect effect**				
IGD → Sleep distress → Depression	0.101 (0.095–0.107)	41.7%	0.011 (0.007–0.014)	21.6%
IGD → Resilience → Depression	0.025 (0.022–0.027)	10.3%	0.004 (0.002–0.006)	7.8%
IGD → Sleep distress → Resilience → Depression	0.021 (0.019–0.023)	8.7%	0.002 (0.001–0.003)	3.9%
**Total indirect effects**	0.147 (0.140–0.153)	60.7%	0.017 (0.012–0.022)	33.3%
**Total effects**	0.242 (0.229–0.254)	-	0.051 (0.037–0.065)	-

Adjusted for baseline demographics (age, sex, family type, residence, family structure, parental education level, left-behind status, only-child status, drinking and smoking), resilience, sleep distress, depression and anxiety symptoms.

The indirect effects of IGD on depression were observed through resilience (*β* = 0.025, 95% CI = 0.022–0.027), through sleep distress (*β* = 0.101, 95% CI = 0.095–0.107) and through the sequential pathway of sleep distress followed by resilience (*β* = 0.021, 95% CI = 0.019–0.023). The total effect of IGD on depression was 0.242 (95% CI = 0.229–0.254), with indirect effects accounting for 60.7% of the total effect – 10.3% through resilience, 41.7% through sleep distress and 8.7% through the sequential pathway of sleep distress to resilience. All paths remained significant after adjusting for baseline characteristics, with a total mediation ratio of 33.3%.

### Sensitivity analysis

Sensitivity analyses were conducted to evaluate the robustness of our findings. First, mediation analyses using participants with complete data across all three waves (*n* = 28,916) were consistent with the primary analysis, with mediation ratios of 59.1% in the unadjusted model and 29.8% in the fully adjusted model (Table S1). Analyses focusing on gamers (*n* = 28,558) also confirmed the serial mediating roles of sleep distress and resilience, with mediation ratios of 61.3% (unadjusted) and 35.4% (adjusted) (Table S2). Treating variables as categorical yielded partial alignment with the main analysis: in the unadjusted model, indirect effects through sleep distress, resilience and their sequential pathway accounted for 64.9% of the total effect, whereas in the adjusted model, only sleep distress remained significant (mediation ratio: 18.8%) (Table S3). Finally, excluding the sleep-related item from the PHQ-9 to reduce shared variance with the PSQI yielded consistent results, with mediation ratios of 59.1% (unadjusted) and 32.7% (adjusted) (Table S4). Collectively, these analyses support the robustness of our findings.

### Sex difference in the mediation analysis

The results indicated a significant sex difference in the mediation model (Δ*χ*^2^ = 98.637, Δ*df* = 6, *p* < 0.001). Table 4 displays the sex-specific differences in the mediation effects of sleep problems and resilience between IGD and depression. In the fully adjusted model, the total mediation ratio was 47.4% in boys and 28.1% in girls, suggesting that sleep distress and resilience may play a more prominent role in the relationship between IGD and depression in boys.

**Table 4.** Sex difference in the mediation effects of sleep problems and resilience between IGD and depression.

	Boys		Girls	
	*β* (95% CI)	Mediation ratio	*β* (95% CI)	Mediation ratio
**Direct effect (IGD → Depression)**	0.020 (0.002–0.038)	-	0.047 (0.031–0.062)	-
**Indirect effect**				
IGD → Sleep distress → Depression	0.004 (0.002–0.006)	10.5%	0.005 (0.002–0.008)	7.8%
IGD → Resilience → Depression	0.012 (0.007–0.017)	31.6%	0.010 (0.006–0.015)	15.6%
IGD → Sleep distress → Resilience → Depression	0.002 (0.001–0.003)	5.3%	0.003 (0.001–0.004)	4.7%
**Total indirect effects**	0.018 (0.012–0.023)	47.4%	0.018 (0.012–0.024)	28.1%
**Total effects**	0.038 (0.020–0.056)	-	0.064 (0.048–0.081)	-

Adjusted for baseline demographics (age, sex, family type, residence, family structure, parental education level, left-behind status, only-child status, drinking and smoking), resilience, sleep distress, depression and anxiety symptoms.

## Discussion

In this large-scale, school-based, three-wave longitudinal study, we explored the direct and indirect effects of IGD on depressive symptoms among Chinese adolescents. The key findings include: (1) IGD was independently associated with depression at baseline (T1: adjusted odds ratio [AOR] = 4.76, 95% CI: 3.79–5.98, *p* < 0.001), 1 year later (T2: AOR = 1.42, 95% CI: 1.16–1.74, *p* < 0.001) and 2 years later (T3: AOR = 1.24, 95% CI: 1.01–1.53, *p* = 0.042); (2) A serial multiple mediation effect of sleep distress and resilience was identified in the relationship between IGD and depression. The mediation ratio was 60.7% in the unadjusted model and 33.3% in the fully adjusted model, accounting for baseline depression, sleep distress, resilience and other covariates; and (3) Gender differences were observed in the mediating roles of sleep distress and resilience, with the mediation ratio being higher in boys compared to girls.

### The direct effect of IGD on depression

To our knowledge, this is the largest cohort study to date investigating the predictive effect of IGD on depressive symptoms. Our findings establish IGD as a significant risk factor for depressive symptoms among adolescents, underscoring the importance of early identification and intervention strategies targeting IGD to prevent depression in this vulnerable population.

Several mechanisms may explain the direct effect of IGD on depression. First, displacement theory suggests that the immersive and competitive nature of online gaming reduces time spent on social interactions, leading to social isolation and diminished social support, both of which increase the risk of depression (Fong *et al.*, 2024; Krossbakken *et al.*, 2018). Second, the intense focus on gaming can result in academic neglect and poor school performance (Hawi *et al.*, 2018), which in turn generates stress from family or teacher conflicts and feelings of inadequacy, further contributing to depression. Third, from a neurobiological perspective, IGD and depression may share similar brain function impairments. For instance, IGD is associated with dysregulation in the brain’s reward system (Weinstein and Lejoyeux, 2020), including heightened sensitivity to gaming-related rewards and diminished responsiveness to other positive stimuli. This imbalance may reinforce gaming as a primary coping mechanism while reducing the ability to derive satisfaction from other life activities, thereby contributing to depression. Structural and functional changes in the prefrontal cortex, a region critical for impulse control, decision-making and emotional regulation, have also been observed in individuals with IGD (Yan *et al.*, 2021). These alterations can impair an adolescent’s ability to manage negative emotions and stress, increasing their vulnerability to depressive symptoms. Choi *et al.* provided direct evidence that lower grey matter density in the left dorsolateral prefrontal cortex mediated the relationship between gaming and depressed mood (Choi *et al.*, 2017). However, much of the current neuroimaging evidence is based on cross-sectional data; therefore, future longitudinal studies are needed to further explore these mechanisms.

### The indirect effect through resilience and sleep distress

Our study is also the first longitudinal investigation into the association between IGD and resilience. In line with findings by Shang *et al.* in the field of internet addiction (Shang *et al.*, 2024), our study demonstrated that resilience serves as a mediator between IGD and depression. This finding suggests that IGD may weaken an adolescent’s resilience, thereby increasing their vulnerability to depression. Individuals with IGD often use gaming as a maladaptive coping strategy, avoiding real-life problems and preventing the development of healthier ways to manage stress (Brand *et al.*, 2016, 2019; King and Delfabbro, 2018). This avoidance behaviour, combined with the emotional instability and cognitive impairments caused by excessive gaming – such as preoccupation with gaming, difficulties in decision-making and self-regulation – further erodes their capacity to adapt to challenges (King and Delfabbro, 2018). The erosion of resilience, in turn, may make adolescents more vulnerable to developing depressive symptoms, as they become less capable of managing the emotional and psychological consequences of their gaming behaviours and other life stressors.

Consistent with previous cross-sectional studies (Alshammari *et al.*, 2022; Li *et al.*, 2019), we found that sleep distress is a key mediator between IGD and depression, accounting for approximately half of the total effects. IGD may contribute to sleep problems through several interconnected pathways, including difficulties in falling asleep, reduced sleep duration and impaired melatonin production due to the blue light emitted by digital devices (Kristensen *et al.*, 2021; Lemola *et al.*, 2015). Importantly, emerging studies suggest a bidirectional relationship between sleep problems and gaming (Liu *et al.*, 2021, 2023; Poulain *et al.*, 2019), indicating a potential vice cycle between the two conditions. Furthermore, sleep problems have been shown to play a crucial mediating role in the relationship between IGD and its various negative psychological consequences (Bersani *et al.*, 2022; Fekih-Romdhane *et al.*, 2023; Garg *et al.*, 2023; Liao *et al.*, 2024). These findings underscore the importance of addressing sleep disturbances in adolescents with IGD to break the cycle of IGD, poor sleep and worsening mental health.

Consistent with Hypothesis 2, we identified a significant indirect effect of IGD on depression through the sequential pathway of sleep distress followed by resilience impairment. This finding suggests that IGD may initially contribute to sleep distress, which subsequently impairs resilience and ultimately leads to depression. This result aligns with prior studies that have identified impaired resilience as a critical mediator between sleep problems and various mental health outcomes, including anxiety symptoms, psychological distress and suicidality (Cai *et al.*, 2023; Jiang *et al.*, 2024; Li *et al.*, 2024). Thus, regular assessment and strengthening of resilience may help mitigate the impact of sleep disturbances on depression.

### Sex difference in the mediating role of resilience and sleep distress between IGD and depression

Our study revealed significant sex differences in the mediating effects of resilience and sleep distress. Despite extensive research on gender differences in IGD and depression, few studies have examined gender differences in the relationship between these conditions and their mediators. Although the exact mechanisms remain unclear, our results suggest that targeting sleep problems and resilience might be particularly effective in boys with IGD to reduce IGD-related depression.

### Clinical implication

Our study offers several key clinical implications. First, the direct and indirect effects of IGD on depression highlight the need for early identification and intervention for IGD to reduce the risk of depression. It is crucial for parents, educators and mental health professionals to be vigilant about the heightened risk of depression linked to IGD and to actively monitor and manage gaming behaviours in adolescents. Second, with one-third of IGD’s impact on depression mediated by sleep distress and impaired resilience, enhancing resilience and improving sleep quality should important intervention strategies. Resilience-building programs, such as cognitive-behavioural therapy focused on coping skills and stress management, combined with promoting healthy sleep practices – like limiting gaming before bed – may be particularly effective in mitigating the effects of IGD on depression (Joyce *et al.*, 2018; Llistosella *et al.*, 2023).

### Strengths and limitations

While our study has several strengths, including a large sample size, unbiased cluster sampling across multiple centres and a longitudinal design, it is not without limitations. First, the reliance on self-reported data for IGD, depression, resilience and sleep distress may introduce biases, such as social desirability and recall bias. Despite the high accuracy and validity of the PHQ-9 and IGDS9-SF, both scales are designed for screening rather than diagnosing depression and IGD. Future studies with clinical interviews are warranted to further validate our findings. Second, although various baseline covariates were controlled for, key factors such as academic stress, childhood trauma and other adverse experiences were not measured, which may have introduced residual confounding. Future studies should include these factors. Third, the generalizability of our findings may be limited, as the study was conducted in a single city in China, and all participants were middle school students. Future research should aim to replicate these findings in adolescents from diverse cultural, educational and clinical settings.

## Conclusion

IGD is predictive of later depression, with sleep distress and impaired resilience acting as key mediators. Addressing IGD might help prevent depression among adolescents. Moreover, targeted interventions that focus on improving sleep quality and enhancing resilience may be effective in mitigating the depression risk associated with IGD, particularly among boys.

## Supporting information

Peng et al. supplementary material Peng et al. supplementary material

## Data Availability

The dataset of the study is available from the corresponding authors upon reasonable request.

## Supplementary material

The supplementary material for this article can be found at https://doi.org/10.1017/S2045796025000046.
