# Supplementary material for: Internet gaming disorder and depression mediated by impaired resilience and sleep distress: a three-wave longitudinal study among Chinese adolescents
Source: Epidemiol Psychiatr Sci. 2025 Feb 19;34:e11. doi: 10.1017/S2045796025000046 (PMC11886973; doi:10.1017/S2045796025000046)
Supplement: Peng et al. supplementary material [file S2045796025000046sup001.docx]

**Supplementary materials for the “Internet gaming disorder and depression mediated by impaired resilience and sleep distress: a three-wave longitudinal study among Chinese adolescents”**

**Table S1. The Mediation effects of sleep and resilience at T2 between T1 IGD and T3 depression in samples who participated in all three waves (n=** **28,916)**

|  | **Unadjusted** | | **Adjusted** | |
| --- | --- | --- | --- | --- |
|  | **β(95%CI)** | **Mediation ratio** | **β(95%CI)** | **Mediation ratio** |
| **Direct effect (IGD → Depression)** | 0.099 (0.087 to 0.111) | - | 0.040 (0.026 to 0.054) | - |
| **Indirect effect** |  |  |  |  |
| IGD →Sleep distress→ Depression | 0.097 (0.091 to 0.103) | 40.1% | 0.010 (0.007 to 0.014) | 17.5 % |
| IGD →Resilience → Depression | 0.025 (0.023 to 0.028) | 10.3% | 0.005 (0.003 to 0.007) | 8.8% |
| IGD →Sleep distress→ Resilience →Depression | 0.021 (0.019 to 0.022) | 8.7% | 0.002 (0.001 to 0.003) | 3.5% |
| **Total indirect effects** | 0.143 (0.136 to 0.149) | 59.1% | 0.017 (0.012 to 0.022) | 29.8% |
| **Total effects** | 0.242 (0.229 to 0.255) | - | 0.057 (0.042 to 0.072) | - |

Adjusted for baseline demographics (age, sex, family type, residence, family structure, parental education level, left-behind status, only-child status, drinking, and smoking), resilience, sleep distress, depression and anxiety symptoms.

**Table S2. The Mediation effects of sleep and resilience at T2 between T1 IGD and T3 depression in gamers (n=** **28,558)**

|  | **Unadjusted** | | **Adjusted** | |
| --- | --- | --- | --- | --- |
|  | **β(95%CI)** | **Mediation ratio** | **β(95%CI)** | **Mediation ratio** |
| **Direct effect (IGD → Depression)** | 0.094 (0.080 to 0.108) | - | 0.031 (0.015 to 0.047) | - |
| **Indirect effect** |  |  |  |  |
| IGD →Sleep distress→ Depression | 0.100 (0.093 to 0.106) | 41.2% | 0.010 (0.005 to 0.014) | 20.8 % |
| IGD →Resilience → Depression | 0.027 (0.024 to 0.031) | 11.1% | 0.005 (0.003 to 0.007) | 10.4% |
| IGD →Sleep distress→ Resilience →Depression | 0.021 (0.019 to 0.024) | 8.6% | 0.002 (0.001 to 0.003) | 4.2% |
| **Total indirect effects** | 0.149 (0.141 to 0.156) | 61.3% | 0.017 (0.011 to 0.023) | 35.4% |
| **Total effects** | 0.243 (0.228 to 0.257) | - | 0.048 (0.031 to 0.064) | - |

Adjusted for baseline demographics (age, sex, family type, residence, family structure, parental education level, left-behind status, only-child status, drinking, and smoking), resilience, sleep distress, depression and anxiety symptoms.

**Table S3. The Mediation effects of sleep and resilience at T2 between T1 IGD and T3 depression (IGD, sleep distress, resilience, and depression as a binary variable)**

|  | **Unadjusted** | | **Adjusted** | |
| --- | --- | --- | --- | --- |
|  | **β(95%CI)** | **Mediation ratio** | **β(95%CI)** | **Mediation ratio** |
| **Direct effect (IGD → Depression)** | 0.051 (0.037 to 0.065) | - | 0.012 (0.002 to 0.023) | - |
| **Indirect effect** |  |  |  |  |
| IGD →Sleep distress→ Depression | 0.025 (0.021 to 0.028) | 47.9% | 0.003 (0.001 to 0.005) | 18.8% |
| IGD →Resilience → Depression | 0.004 (0.003 to 0.006) | 5.3% | 0.000 (-0.001 to 0.001) | - |
| IGD →Sleep distress→ Resilience →Depression | 0.004 (0.003 to 0.005) | 11.7% | 0.000 (0.000 to 0.001) | - |
| **Total indirect effects** | 0.033 (0.029 to 0.037) | 64.9% | 0.003 (0.001 to 0.006) | 18.8% |
| **Total effects** | 0.084 (0.069 to 0.099) | - | 0.016 (0.005 to 0.026) | - |

Adjusted for baseline demographics (age, sex, family type, residence, family structure, parental education level, left-behind status, only-child status, drinking, and smoking), resilience, sleep distress, depression and anxiety symptoms.

**Table S4. The Mediation effects of sleep and resilience at T2 between T1 IGD and T3 depression (Excluding sleep-related items in PHQ9)**

|  | **Unadjusted** | | **Adjusted** | |
| --- | --- | --- | --- | --- |
|  | **β(95%CI)** | **Mediation ratio** | **β(95%CI)** | **Mediation ratio** |
| **Direct effect (IGD → Depression)** | 0.098 (0.086 to 0.111) | - | 0.037 (0.024 to 0.051) | - |
| **Indirect effect** |  |  |  |  |
| IGD →Sleep distress→ Depression | 0.097 (0.091 to 0.102) | 40.1% | 0.011 (0.007 to 0.015) | 20.0 % |
| IGD →Resilience → Depression | 0.026 (0.023 to 0.029) | 10.3% | 0.004 (0.002 to 0.006) | 7.3% |
| IGD →Sleep distress→ Resilience →Depression | 0.022 (0.020 to 0.024) | 8.7% | 0.002 (0.002 to 0.003) | 3.6% |
| **Total indirect effects** | 0.144 (0.138 to 0.151) | 59.1% | 0.018 (0.013 to 0.022) | 32.7% |
| **Total effects** | 0.243 (0.230 to 0.255) | - | 0.055 (0.041 to 0.069) | - |

Adjusted for baseline demographics (age, sex, family type, residence, family structure, parental education level, left-behind status, only-child status, drinking, and smoking), resilience, sleep distress, depression and anxiety symptoms.
